# A Rare Case of Kikuchi Fujimoto's Disease with Subsequent Development of Systemic Lupus Erythematosus

**DOI:** 10.1155/2012/325062

**Published:** 2012-12-30

**Authors:** Yu Zuo, Michelle Foshat, You-wen Qian, Brent Kelly, Brock Harper, Bernard Karnath

**Affiliations:** Department of Internal Medicine, University of Texas Medical Branch, 301 University Boulevard, Galveston, TX 77555, USA

## Abstract

Kikuchi Fujimoto's disease (KFD) is a rare, immune-mediated, self-limiting disorder with unique histopathological features. KFD is usually seen in young Asian females; however, cases have been reported throughout the world and in all ethnicities. It has been recognized that there is a rare association between Systemic Lupus Erythematosus (SLE) and KFD via sporadic case reports. The exact pathophysiological relationship between these two diseases is still unclear. We report a case of a young Asian female who presented with persistent fever and lymphadenopathy and was diagnosed with Kikuchi Fujimoto's disease based on lymph node biopsy; although an SLE workup was done, she did not meet the American Rheumatology Association (ARA) diagnostic criteria for lupus, and the lymph node biopsy did not show features of SLE. She improved clinically with a short course of steroid therapy. Two months later, the patient presented with central facial rash and arthralgia. SLE workup was repeated, a skin biopsy was done, and the results at this time supported a diagnosis of SLE.

## 1. Introduction

Kikuchi Fujimoto's disease (KFD) is a rare, immune-mediated, typically self-limiting disease first described in Japan in 1972 [1, 2]. It has been seen in all ethnic groups around the world; however, most cases are reported in Asia and the Far East. Between 1991 and 2005, there were a total of 330 cases of KFD reported in Medline database around the world, and only 22 cases were reported in the US [3]. Clinically, patients present with persistent fever and lymphadenopathy which may be painful; cervical lymph nodes are most commonly involved. Other symptoms such as fatigue, night sweats, nausea and vomiting, weight loss, arthralgias, and a variety of cutaneous lesions are also reported. Laboratory findings are nonspecific including elevated Erythrocyte Sedimentation Rate (ESR), neutropenia, lymphocytosis, mildly elevated transaminase, and elevated Lactate Dehydrogenase (LDH) [3–5]. Diagnosis is made via lymph node biopsy with a histopathological finding characterized by a histiocytic necrotizing lymphadenitis without granulocytic infiltrate [3]. In most cases, KFD is a self-limiting disease; only supportive treatment is required. However, case reports suggest that a short course of corticosteroids can expedite remission. The association of Systemic Lupus Erythematosus (SLE) and KFD is even rarer; out of 330 cases reported between 1991 and 2005, only 28 patients with KFD were also diagnosed with SLE [3]. By Medline search, between 2005 and 2012, there were only 9 more additional SLE-associated KFD cases reported around the world [6–14].

## 2. Case Presentation

**Figure 1:**
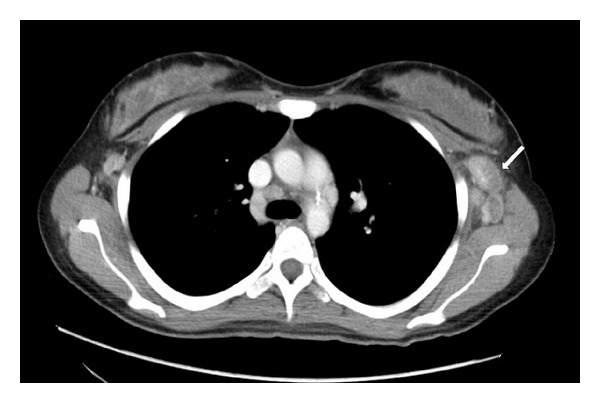
Axillary lymphadenopathy on CT thorax with contrast.

**Figure 2:**
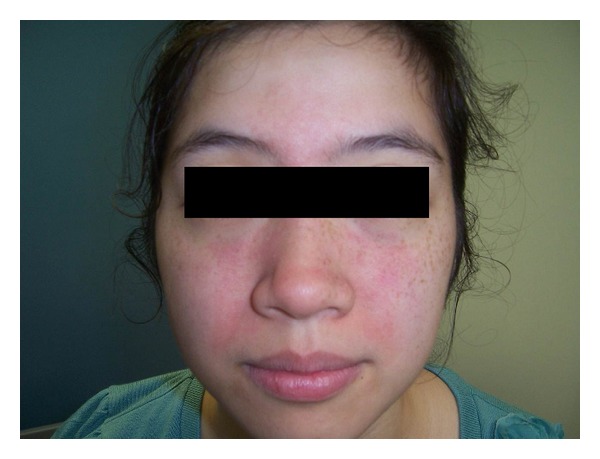
Patient presents with central facial rash 2 months after the diagnosis of KFD.

A 23-year-old Asian previously healthy woman presented with persistent fever and cervical, supraclavicular, and axillary lymphadenopathy. She denied chest pain, SOB, nausea, vomiting, weight loss, dizziness, diarrhea, urinary symptoms, rash, joint pain, headache, or vaginal discharge. She denied sick contacts, recent travel, exposure to pets/animals, bug bites, new medications, and herbal supplements. Prior to admission, the patient was evaluated at a community urgent care center where she had negative monospot test, Epstein-Barr Virus (EBV) PCR, and Cytomegalovirus (CMV) IgG. Upon admission, pt had a fever of 102.7, tachycardia at 115, and BP 109/74. Physical exam showed diffuse, rubbery, mobile, and tender lymphadenopathy of bilateral anterior cervical lymph nodes, bilateral axillary lymph nodes, and left supraclavicular lymph nodes. No rash was seen on her skin. There was no joint tenderness, warmness, erythema, or swelling. Cardiovascular and respiratory exams were otherwise unremarkable. CT scan showed enlarged axillary lymph nodes with partially necrotic appearing centers (Figure 1), partially visualized supraclavicular lymphadenopathy, and borderline to mildly enlarged mediastinal lymph nodes. Labs revealed WBC 3700, Antinuclear Antibody (ANA) positive with a titer of 1 : 80, Rheumatoid Factor low positive at 21 IU/mL (normal at 20 IU/mL), anti-dsDNA at 118 u/mL at equivocal range, ESR elevated to 110 mm/hr, elevated C-reactive protein (CRP), Toxoplasma IgG and IgM negative, Parvovirus IgM negative, SSA and SSB negative, and PPD negative. Core lymph node biopsy showed no increased neutrophils, eosinophils, or plasma cells. No typical microscopic signs of vasculitis or Hematoxylin bodies were noted. AFB for mycobacteria, GMS for fungi, and Warthin-Starry for Cat-Scratch disease were all negative. Overall features were consistent with histiocytic necrotizing lymphadenitis. Based on the previous pathological finding, a diagnosis of Kikuchi Fujimoto's disease was made. The patient was given prednisone 40 mg daily; after the first dose, her fever resolved and symptoms improved. She received two doses of prednisone in the hospital and was discharged with an additional 5 days of prednisone 40 mg daily. The patient developed a central facial erythematous patch with scaling (Figure 2) one month after discharge. She denied joint pain or swelling, fever, and fatigue; lymphadenopathy had significantly reduced in size. She was referred to dermatology and was diagnosed with seborrheic dermatitis and discharged home with desonide 0.05% cream and ketoconazole cream 2%. She was also evaluated by a rheumatologist; at that time she did not have the complete picture of SLE. Two months after discharge, she was seen again at the Rheumatology clinic with mild elbow swelling and pain. Physical exam showed small effusion with inability to fully extend her elbows bilaterally. The patient was also seen in the dermatology clinic that same day, with some improvement of her facial rash but with new erythematous patches with scaling on her scalp. A punch skin biopsy was obtained at that time. Labs at that time revealed that CBC, CRP, and CMP were unremarkable. Urinalysis did not show proteinuria, pyuria, or bacteria. ANA was positive with significant IFA titer of 1 : 320, complement C3 and C4 were decreased, and SSA positive, SSB negative, anti-ds DNA positive at 233.6 u/mL, anti-Smith positive at 79.5 units, and anti-ribonucleic protein (RNP) antibodies positive at 113.9 units were evident. These labs were consistent with SLE. The skin punch biopsy showed hyperkeratosis, follicular plugging, interface dermatitis, patchy perivascular, and periadnexal lymphocytic infiltrate with a focal area of increased dermal mucin consistent with discoid lupus erythematosus (Figure 4). She was diagnosed with SLE and placed on hydroxychloroquine.

## 3. Discussion

KFD is a rare, benign, self-limiting, immune-mediated disorder that usually resolves spontaneously after 1–4 months [15]. It occurs more frequently among young Asian females; however, cases had been reported in all continents and among all ethnic groups [16]. Based on a study done by Kucukardali et al., in 2007, 330 cases of KFD were reported in the Medline database; among those, 77% were females with a mean age of 25, and 70% of patients were under age of 30. Asia had the most reported cases followed by Europe and America. The most common symptoms were persistent fever and lymphadenopathy. High sedimentation rate is observed in a majority of patients, and positive ANA has also been reported in KFD patients. Moreover, 64% of patients improved without any treatment, 16% of patients received steroid treatment and the rest had various other treatments [3, 17]. Sporadic SLE-associated KFD cases have been reported, and the exact clinical and pathophysiological relationships between these two diseases are not known. SLE-associated KFD cases are more common in Asian countries. Among those cases, SLE has been seen to either coincide, proceed, or follow a diagnosis of KFD [16]. Kucukardali et al. reported 28 cases of SLE-associated KDF that meet the diagnostic criteria for SLE. Eighteen patients were diagnosed with KFD and SLE simultaneously, four had a previous diagnosis of SLE, and six were diagnosed with SLE after they were diagnosed with KFD [3]. Santana et al., in 2005, reported a total of 35 cases SLE-associated KFD all over the world based on Medline and LILACS databases. Seven patients were diagnosed with SLE prior to KFD diagnosis, fourteen patients were diagnosed with KFD and SLE simultaneously, and fourteen patients were diagnosed with SLE after a diagnosis of KFD [15]. Clinical features of SLE and KFD can be very similar; fever, lymphadenopathy, fatigue, and joint pain can be seen in both diseases. Definite discrimination between those two diseases is based on histopathological findings; the presence of hematoxylin, plasma cells, and deposition of DNA is seen in SLE patients but not in KFD patients. Kim et al. had found that SLE-associated KFD patients tend to have high incidence of skin manifestations [16]. 

**Figure 3:**
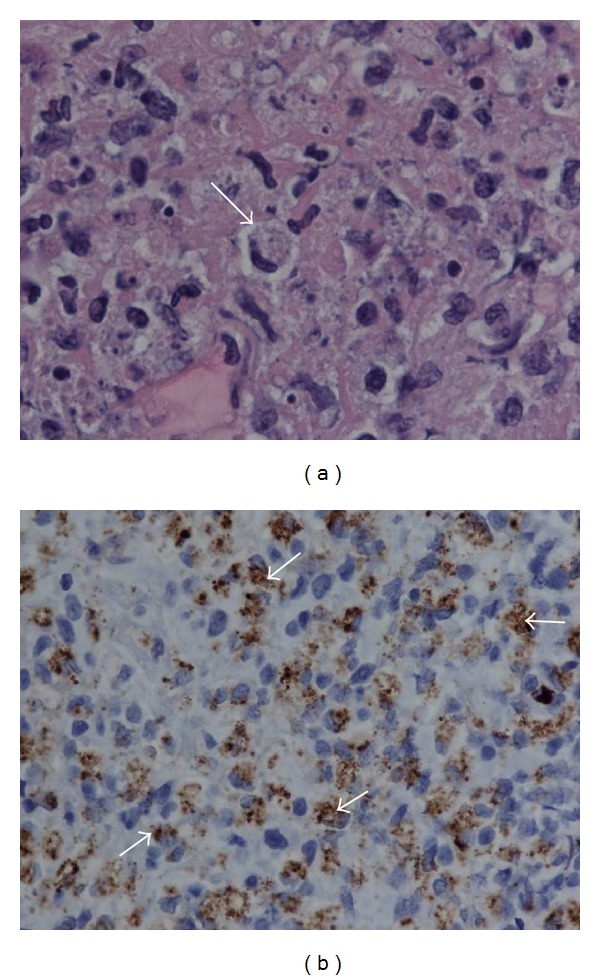
(a) Crescent histiocyte (arrow) in a background of karyorrhectic debris and histiocyte proliferation, 1000x. (b) Numerous histiocytes show cytoplasmic staining for MPO, 500x.

**Figure 4:**
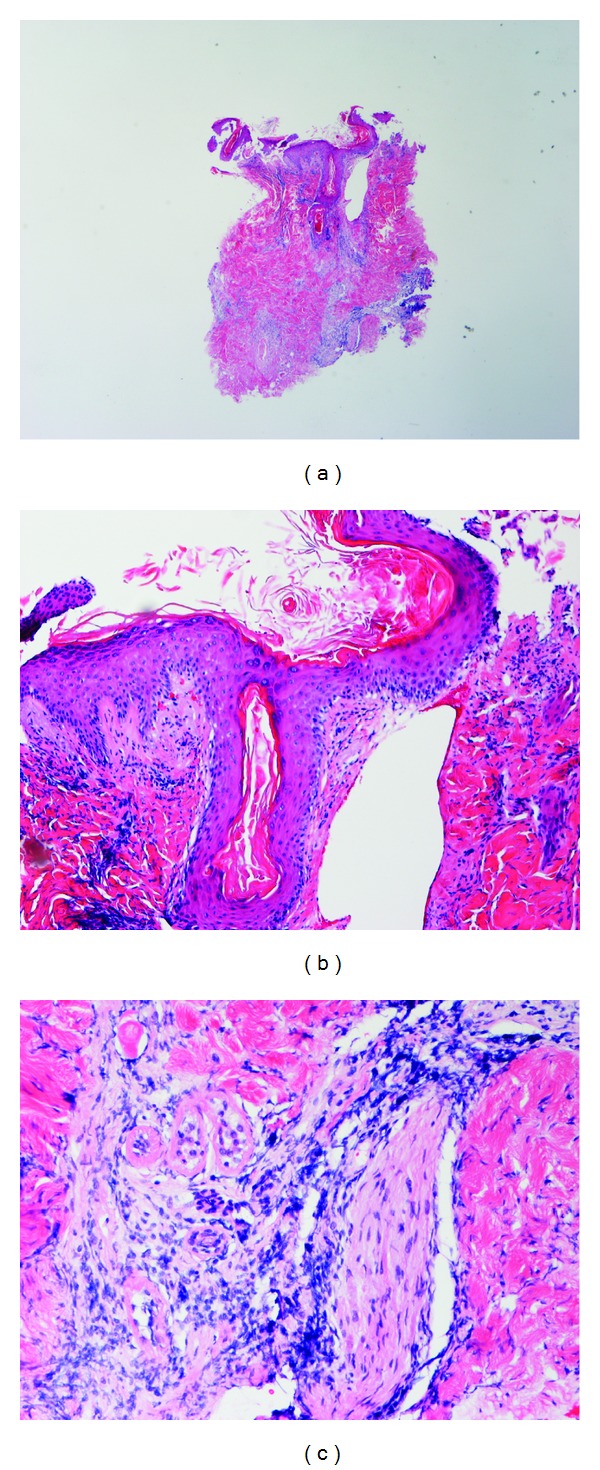
(a), (b), and (c) The skin punch biopsy showed hyperkeratosis, follicular plugging, interface dermatitis, and patchy perivascular and periadnexal lymphocytic infiltrate with a focal area of increased dermal mucin consistent with discoid lupus erythematosus.

Our patient fits into the epidemiological traits of KFD and has a typical KFD clinical presentation. She is a young, Asian, previously healthy female who presented with persistent fever and lymphadenopathy. No joint pain or skin manifestation was present initially. Infectious disease workup has all been negative. ESR was elevated. She did have a positive ANA but with a low titer. Anti-dsDNA was in the equivocal range. SSA and SSB were negative. Lymph node biopsy shows partially preserved lymphoid architecture with rare lymphoid follicles and extensive coagulative necrosis. The necrotic areas contain abundant karyorrhectic debris. The necrosis is often surrounded by and intermingled with many histiocytes that occasionally contain engulfed nuclear debris with the nuclei pushed to the periphery giving the nuclei crescent appearances (Figure 3(a)). The histiocytes, highlighted by CD68, demonstrate myeloperoxidase (MPO) positivity (Figure 3(b)). Noted in the cellular area are some large cells with prominent nucleoli and amphophilic cytoplasm, morphologically mostly consistent with plasmacytoid dendritic cells. Many small CD3 positive T-lymphocytes are scattered in the necrotic areas with CD8 positive T-cells outnumbering CD4 positive T-cells. CD20 shows few residual lymphoid follicles. The overall findings are that of a Kikuchi Hashimoto lymphadenopathy [18–20]. The CD20 and CD3 staining results do not support a diagnosis of B- or T-cell lymphoma. Due to the fact that there are no increased neutrophils, eosinophils, or plasma cells, and vasculitis and hematoxylin bodies are not noted in the biopsy, a diagnosis of lupus lymphadenitis is not suggested pathologically. 

She did not meet the diagnosis criteria for SLE upon admission. History, clinical, laboratory, and biopsy findings all support a diagnosis of KFD. Two months later, the patient presented with malar rash, joint pain, positive ANA at high titer, increased anti-ds DNA, and positive anti-RNP antibodies; at that point, the patient met the American Rheumatology Association diagnostic criteria for SLE. Her skin core biopsy finding is consistent with discoid lupus erythematosus which further confirms a diagnosis of SLE.

In conclusion, although KFD is a rare and self-limiting condition, it is important to recognize this condition in a patient who fits the epidemiological background with typical clinical features to avoid burdensome investigative workup and adding unnecessary anxiety to patients. In addition, when managing patient with KFD, it is crucial to recognize the relationship between SLE and KFD and carry out careful clinical surveillance to identify potential coexistence of SLE and to monitor for future development of SLE.
